# Sudden deaths of two dogs in a breeding colony associated with enteropathogenic *Escherichia coli* and necrotoxigenic *Escherichia coli*

**DOI:** 10.1128/asmcr.00064-24

**Published:** 2025-08-25

**Authors:** Maho Okumura, Nathan Helgert, Katharine Tuohy, Donna J. Kelly, Stephen D. Cole

**Affiliations:** 1 University of Pennsylvania School of Veterinary Medicine 6572, Philadelphia, Pennsylvania, USA; 2 University of Pennsylvania School of Veterinary Medicine 6572, Kennett Square, Pennsylvania, USA; Pattern Bioscience, Austin, Texas, USA

**Keywords:** *Escherichia coli*, EPEC infection in canines, ExP-NTEC in canines, canine sudden death

## Abstract

**Background:**

This case report describes the peracute deaths of two healthy pups in a breeding facility associated with enteropathogenic *Escherichia coli* (EPEC) and a distinct strain of necrotoxigenic *E. coli* (ExP-NTEC) co-infection. When *E. coli* is recovered from post-mortem samples, the role of the organism can be challenging to elucidate. In this case, the use of whole-genome sequencing (WGS) uncovered the virulence factors associated with isolates from both cases and a molecular epidemiological connection between the two deaths.

**Case Summary:**

A 7-week-old male and an 8-week-old female intact mixed-breed dogs were found deceased, shortly after routine examination with no preceding clinical signs. Post-mortem examination revealed multiple organ abnormalities, including bronchopneumonia with alveolar collapse, hepatic lipidosis, gastrointestinal inflammation, and bacterial colonization in the lungs and intestines. Bacterial cultures from the lungs and intestines of both pups yielded multiple bacterial species. WGS of six *E. coli* isolates from both pups identified ST5683 and ST127 strains, possessing two different morphotypes and virulence factors that distinguished them as EPEC ExP-NTEC.

**Conclusion:**

The findings suggest a role of EPEC and ExP-NTEC in the sudden deaths of neonatal dogs, possibly exacerbated by a naïve immune system. It is challenging to fully characterize the pathogenesis and potential risk factors as *E. coli* is present in the gastrointestinal system of healthy and asymptomatic carrier animals; however, these cases highlight the importance of comprehensive diagnostic approaches, including tools such as WGS.

## INTRODUCTION

*Escherichia coli* is an important gram-negative bacterium that is a normal inhabitant of many species’ gastrointestinal systems. Although many *E. coli* strains are not considered pathogenic, some lineages are associated with a wide variety of intestinal and extraintestinal diseases in humans and animals. Intestinal pathogenic *E. coli* species have been grouped into several major pathotypes, including enteropathogenic *E. coli* (EPEC), enterotoxigenic *E. coli*, enteroinvasive *E. coli*, and Shiga-toxin-producing *E. coli*/enterohemorrhagic *E. coli* (1). These are distinct from extraintestinal pathogenic *E. coli* (ExPEC), which tend to cause infections outside of the gastrointestinal tracts, including uropathogenic, neonatal meningitis, and necrotoxigenic *E. coli* (NTEC). Knowledge of specific virulence factors produced by bacterial isolates may enhance our understanding of the potential role isolates play in the clinical manifestation of disease. Whole-genome sequencing (WGS) has emerged as a powerful tool to aid in these investigations. This case report describes the investigation into the sudden deaths of two apparently healthy pups in a breeding facility.

## CASE PRESENTATION

### Patient information

The animals were one 7-week-old intact male and one 8-week-old intact female mixed-breed dogs from different litters and parents, born 11 weeks apart, and were deemed apparently healthy after a routine veterinary examination. They were bred as models to study congenital disease. Both pups received a combination vaccine for canine distemper virus, canine adenovirus-2, canine parvovirus, and canine parainfluenza virus, as well as routine fenbendazole (50 mg/kg) for deworming at 6 weeks of age.

### Clinical findings

The pups were found deceased without premonitory signs, 3 months apart, and without prior symptoms, despite normal health checks hours earlier. All other littermates and dogs in the same facility were apparently healthy. The bitches were also apparently healthy. The pups had ideal body condition scores (5/9) with no other clinical issues reported other than mild loose stool in the female pup. Both pups were negative for canine parvovirus on fecal point-of-care antigen testing (SNAP Parvo Test, IDEXX Veterinary Diagnostics, Westbrooke, ME).

### Diagnostic assessment

#### Gross post-mortem examination

At the time of postmortem examination, the male pup had diffusely white mucous membranes, and the right superficial cervical lymph node was mildly enlarged. The left lung lobes were light pink with subtle rib impressions. The right caudoventral lung was dark red and firm, consistent with regional bronchopneumonia. A representative section of the small intestines was saved in a freezer at −20°C as part of the routine sampling for further investigation. The female pup’s stomach contained green-brown mucoid material, and the small intestines contained a mild amount of opaque white fluid. The lungs were diffusely soft and light red. A representative section of the lung and small intestines was separately saved in a freezer at −20°C. Both dogs had evidence of hepatic lipidosis, while other organs appeared grossly normal.

#### Histopathology

Samples of major organs were fixed in 10% neutral buffered formalin, routinely embedded in paraffin, sectioned, and stained with hematoxylin and eosin or McDonald Gram stain.

**Fig 1:**
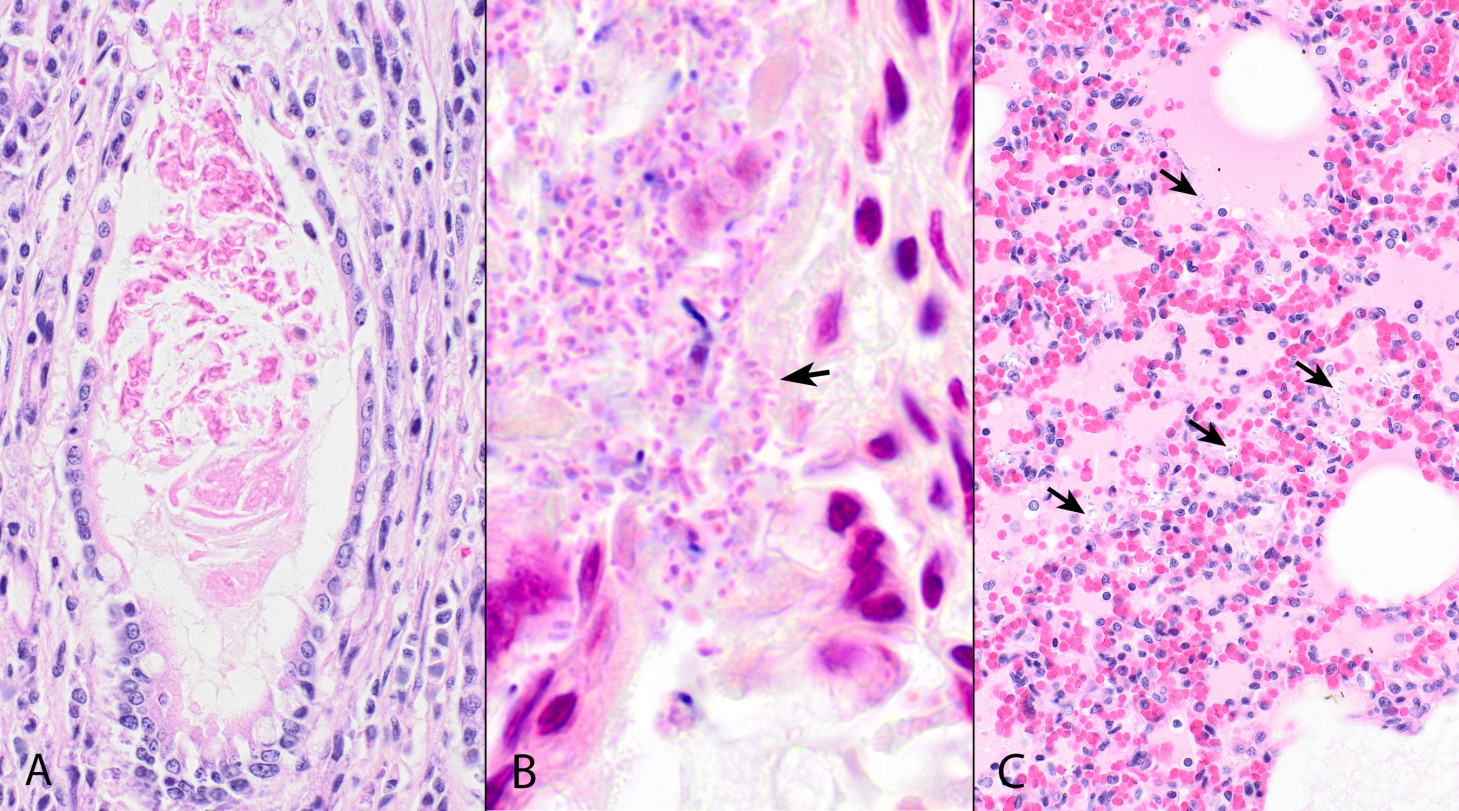
Histopathological findings. (A) Jejunum, female puppy. Occasional crypts were moderately to markedly ectatic and filled with cellular debris (crypt ectasia). Original magnification 400×. Hematoxylin and eosin. (B) Small intestine, male puppy. Within the brush border of enterocytes are numerous gram-negative coccobacilli and short bacilli (arrow). Original magnification 1,000×. McDonald's modified Gram stain. (C) Lung, male puppy. Alveoli contain bacteria (arrows), homogeneous, lightly eosinophilic material, and scant inflammatory cells. Original magnification 400×. Hematoxylin and eosin.

Within the small and large intestines of the male pup were mild multifocal crypt ectasia, lymphoid depletion, and abundant gram-negative short bacilli within the brush border (Fig. 1B). In the lungs, alveoli were collapsed (atelectasis) or contained light eosinophilic fluid, mixed morphology bacteria, squamous epithelial cells, and mild numbers of neutrophils and macrophages (Fig. 1C). In the female pup, the duodenum was infiltrated by mild numbers of lymphocytes and plasma cells, and occasional crypts were ectatic and filled with amorphous eosinophilic material (Fig. 1A), and within the brush border of the jejunum and ileum were numerous gram-negative short bacilli. Alveoli contained neutrophils, mixed morphology bacteria, and protein. Apart from confirmation of hepatic lipidosis, other tissues examined were free from histological abnormalities.

#### Bacteriology

Swabs of thawed lung and intestines from the female pup and intestines from the male pup were collected after the suspicion of an epidemiological link between the cases was established and submitted for aerobic culture. The swabs were streaked on Tryptic Soy with 5% sheep blood, MacConkey, and Columbia Colistin-Nalidixic Acid agar plates (Remel, Lenexa, KS) and incubated for 24–48 hours at 35°C–37°C in 7% carbon dioxide. The sample from the lung was also streaked on chocolate agar (Remel, Lenexa, KS). All bacterial colonies were identified using matrix-assisted laser desorption ionization time of flight (Sirius 1 Biotyper, Bruker, Billerica, MA). *E. coli* with two different morphologies was isolated from each swab (for a total of six *E. coli* isolates) along with other bacteria detailed in Table 1.

**TABLE 1:** Results of aerobic culture from the two patients^*a*^^,^^*b*^

Patient	Source	*E. coli* morphotypes	*E. coli* lactose fermentation	*E. coli* sequence type (Achtman)	*E. coli* serotype	Other organisms identified
Male pup	Intestine	Beta hemolytic, irregular, dull, spreading, and gray colonies (1+)	Lactose fermenter	ST5683	O115:H25	*Enterococcus faecalis* (3+) *Enterococcus hirae* (2+) *Staphylococcus pseudintermedius* (scant)
		Round, raised, glistening, and gray colonies (1+)	Non-lactose fermenter	ST127	O6:H31	
Female pup	Lung	Beta hemolytic, irregular, dull, spreading, and gray colonies	Lactose fermenter	ST5683	O115:H25	*Proteus sp*. *Streptococcus canis* *Lactococcus garvieae* *Enterococcus faecalis*
		Round, raised, glistening, and gray colonies	Non-lactose fermenter	ST127	O6:H31	
	Intestine	Beta hemolytic, irregular, dull, spreading, and gray colonies	Lactose fermenter	ST5683	O115:H25	*Proteus sp*. *Streptococcus canis* *Enterococcus faecalis* *Enterococcus hirae* *Vagococcus sp*.
		Round, raised, glistening, and gray colonies	Non-lactose fermenter	ST127	O6:H31	

^*a*^ 1+ stands for scant growth characterized by growth in the first quadrant. 2+ stands for light growth characterized by growth in the first and second quadrants. 3+ stands for moderate growth characterized by growth in the first, second, and third quadrants.

^*b*^ Enumeration could not be performed on the cultures from the female pup samples due to *Proteus* swarm on Tryptic Soy agar with 5% sheep blood.

### Whole-genome sequencing

Six *E. coli* isolates were sequenced: two intestinal and two pulmonary isolates from the female pup and two intestinal isolates from the male pup. Short-read sequencing was performed on the NovaSeq X Plus sequencer (Illumina). Libraries were prepared using the Illumina DNA Prep Kit and custom IDT 10 bp unique dual indices (280 bp). Demultiplexing, quality control, and adapter trimming were performed using a proprietary Illumina software. Draft genomes were assembled using the default parameters of the Bacterial and Viral Bioinformatics Resource Center (BV-BRC) Comprehensive Genome Analysis pipeline. Assemblies were then analyzed using *in silico* MLST, SeroTypeFinder, and VirulenceFinder applications (1–11). Raw sequence reads (NCBI Bioproject PRJNA1213789) were processed via the Pathogen Genome Annotation Pipeline (12–14), which includes phylogenetic analysis to identify database isolates within 50 single-nucleotide polymorphisms (SNPs) (15, 16).

The six isolates represented a total of two strains of *E. coli*: ST127 with serotype O6:H31 and ST5683 with serotype O115:H25. One of each detected strain was found in each specimen. The isolates from all sources were found to be very closely related, with the three ST127 isolates from the two dogs being 1 to 2 SNPs apart and the three ST5683 isolates 0 to 1 SNPs apart. The virulence factors that were detected are summarized in Table 2.

**TABLE 2:** Virulence factors detected from the two strains of *E. coli* upon VirulenceFinder analysis (Center for Genomic Epidemiology)

Virulence factor	% Identity	Function
ST127		
*cnf1*	100	Cytotoxic necrotizing factor 1
*fimH*	100	Type 1 fimbriae
*kpsMII*	100	Polysialic acid transport protein, group 2 capsule
*traT*	100	Outer membrane protein complement resistance
*terC*	99.72	Tellurium ion resistance protein
*yehA/B/C/D*	95.21–97.68	YHD fimbrial cluster
ST5683		
*bfpA*	100	Bundle-forming protein subunit
*fimH*	100	Type I fimbriae
*eae-e01-epsilon*	99.89	Intimin gene variant
*traT*	99.86	Outer membrane protein complement resistance
*astA*	98.97	Heat-stable enterotoxin EAST-1

### Therapeutic intervention

No clinical concerns were observed in the other animals in the colony; therefore, prophylactic treatment was not administered.

### Follow-up and outcomes

There were no additional deaths in the colony following these two cases.

## DISCUSSION

This case highlights the sudden deaths of two otherwise healthy pups in a breeding colony associated with EPEC and NTEC infection. In veterinary clinical microbiology, we are often asked to contribute to “herd health” investigations, which often rely on post-mortem examination to characterize deaths; however, specimens collected post-mortem are often non-ideal for bacteriology and may yield difficult-to-interpret results. For example, in this investigation, the two cases were separated by approximately 3 months, which led to the submission of limited frozen specimens. While we acknowledge these specimen limitations and the need for cautious interpretation, *E. coli* infection was determined to be a likely diagnosis based on clinical history, bacteriology, and pathology results. We added additional evidence by further characterizing the *E. coli* isolates with WGS to determine relatedness and pathogenic potential of isolates.

We believe it is reasonable to conclude that both puppies had gastrointestinal disease that led to vomiting or regurgitation and subsequent aspiration pneumonia. In both cases, bacterial colonization of the small intestinal brush border was suggestive of EPEC infection. EPEC pathogenesis is characterized by the destruction of the microvilli from intimate adherence to the intestinal epithelium, pedestal formation, and aggregation of polarized actin and other cytoskeleton elements (17). In these cases, EPEC infection was confirmed by WGS identification of EPEC-associated virulence factors (*bfpA*, *eae*-e01-epsilon, and *tir*) in the ST5683 isolates. Our EPEC isolates were serotype O115, which is not one of the 12 serotypes described by the WHO, but *E. coli* O115 has been previously described as an atypical EPEC (17–21).

The aspiration pneumonia was most likely polymicrobial in nature based on histopathology and bacteriology results, which included isolation of both *E. coli* and *Streptococcus cani*s, which are commonly associated with aspiration bacterial pneumonia in dogs (21). Specifically, in both dogs, we detected *E. coli* ST127, which was classified as both ExPEC and NTEC by WGS. ST127 has been previously detected in a young dog presenting with vomiting, lethargy, and labored breathing due to a hemothorax, severe hemorrhagic pneumonia, and hemorrhagic enteritis (22, 23). *E. coli* ST127 has previously been cultured from the lung and small intestines of dogs, minks, and humans who have succumbed to hemorrhagic and non-hemorrhagic pneumonia and sepsis (24–26). Virulence factors found in the sequenced isolates included *fimH*, *sitA*, *kpsMII*, and t*raT*, consistent with ExPEC and *cnf*1, consistent with NTEC (27, 28). *Cnf1* activates the regulatory Rho, Rac, and Cdc42 GTPases in eukaryotic cells, leading to actin cytoskeletal rearrangement and cellular damage (29).

Given the polymicrobial nature of the cultures, it is not feasible to fully characterize to what degree the *E. coli* relative to other bacteria contributed to disease pathogenesis. Additionally, it is known that in some mammals, EPEC and ExPEC can asymptomatically colonize the gastrointestinal tract, so we cannot fully exclude the possibility of these bacteria being incidental findings in our investigation when isolated from the gastrointestinal tract (30, 31).

The isolates from both dogs were found to be closely related to each other by SNP analysis, suggesting a close epidemiological link. In animal colony settings, biosecurity measures including quarantine and isolation of new or sick animals, use of personal protective equipment by staff, and strict cleaning and disinfection must be used to prevent the spread of pathogens. The isolated *E. coli* may be part of the normal gastrointestinal flora of an adult dog, which may pose unique challenges for animal care staff when compared to overt pathogens to combat. As these cases were separated by time, further biosecurity interventions were not pursued; however, if future cases were to occur, this investigation has provided valuable information on the potential role of *E. coli* in these deaths.
